# Real-Time Depth-Based Hand Detection and Tracking

**DOI:** 10.1155/2014/284827

**Published:** 2014-03-09

**Authors:** Sung-Il Joo, Sun-Hee Weon, Hyung-Il Choi

**Affiliations:** Department of Media, Soongsil University, Seoul 156-743, Republic of Korea

## Abstract

This paper illustrates the hand detection and tracking method that operates in real time on depth data. To detect a hand region, we propose the classifier that combines a boosting and a cascade structure. The classifier uses the features of depth-difference at the stage of detection as well as learning. The features of each candidate segment are to be computed by subtracting the averages of depth values of subblocks from the central depth value of the segment. The features are selectively employed according to their discriminating power when constructing the classifier. To predict a hand region in a successive frame, a seed point in the next frame is to be determined. Starting from the seed point, a region growing scheme is applied to obtain a hand region. To determine the central point of a hand, we propose the so-called Depth Adaptive Mean Shift algorithm. DAM-Shift is a variant of CAM-Shift (Bradski, 1998), where the size of the search disk varies according to the depth of a hand. We have evaluated the proposed hand detection and tracking algorithm by comparing it against the existing AdaBoost (Friedman et al., 2000) qualitatively and quantitatively. We have analyzed the tracking accuracy through performance tests in various situations.

## 1. Introduction

In the past decade, there have been intensive studies on the automatic analyses of human behaviors. Among the study areas, the human-computer interaction field has attracted the most attention, and there have been many studies on human gesture recognition. A gesture is an effective nonverbal communication tool that helps in complex human interactions with its ability for simple communication. Hand gesture recognition is applied to many fields from a sign language system for the hearing impaired to smart devices for effective interactions. Various gesture recognition approaches that involve hand region detection, hand feature extraction, and learning and recognition methods have been reported. The existing studies include the use of a data glove to analyze hand images [1–3], color data [4, 5], combination of color and depth data [6–8], and depth data alone [9–13]. The use of a data glove is limited and difficult to build an easy interface because of its requirement of a connecting line to connect to the entire system.

The methods that use color images use such information as skin color or edges. Suk and Sin [4] detect a face and hand region using Haar-like features and combined the skin color model. The detected region is tracked using a Gauss function and recognized by the tracked path. The drawbacks of this method are that it requires a preceding condition, that is, face detection prior to hand detection, and that it is sensitive to light changes. Bhuyan et al. [5] detect a hand region using the distribution of skin colors in the RGB space and the conditional probabilities of the foreground and the background. The hand and arm regions are divided by detecting the center point and the main direction of the hand from the detected hand region, and the fingertip is detected on the basis of the geometric features of the hand region. However, this method has the following drawbacks: (1) it detects the hand region by using the existing skin color model. (2) Its experimental environment is very limited. (3) It is sensitive to the occlusion of the hand region with object neighbors.

Many studies that combine it with depth data have been carried out to nullify the weakness of the color data method with respect to environmental changes. Park et al. [6], under the assumption that the hand lies before the body, draw an accumulated histogram from a depth image of Kinect to find the candidate hand regions and detect a final hand region by using Bayes' rule and the skin color to find the precise hand region. This method performs considerably better than the sole use of the color and depth data, but performance decreases in darkness because of its basic assumption; that is, the hand always lies before the body and the use of color. Van den Bergh and Van Gool [7] proposed a mixed method; that is, it detects a face from the RGB image, deletes the background by applying the threshold value of the distance of the detected face, and then detects the hand region from the remaining region. This method also is more precise than the methods that use either a color or a depth image but needs more computation and cannot be used under dim lighting. Trindade et al. [8] perform skin color filtering by using the RGB color from the RGB-D sensor prior to detecting the body, face, and hand regions, distribute the histogram according to the depth axis, and filter out the hand region on the basis of a threshold value. Then, the outliers are deleted by *k*-means clustering to detect the center point of the hand region, which becomes the base point for hand region detection and pose recognition. This method, with a combined use of color and depth data, could improve precision in the detection process by removing outliers during filtering and applying a clustering technique. However, it is weak with respect to the change of lighting and is vulnerable to errors as it goes through several processes until the hand region detection.

Although the methods that combine color and depth data improve the detection of hand region, they are still limited because of the color dependability. There are also studies that did not require color data and used only depth data. Mo and Neumann [9] define a hand model to recognize a figure of a hand at a low resolution and use the depth data inputted from a laser-based camera. They assume that the closest region from the camera is the hand of a user and then divide the hand, wrist, and background regions. However, it fails to detect these regions when the hand is positioned behind the body or there is an object between the camera and the hand. Liu and Fujimura [10] assume an object within a certain distance from the camera as a human and detect the face by horizontal and vertical projections. For the gesture recognition, it is assumed that the hands are in general apart from the body; therefore, the hand regions are detected by using a proportionate constant to separate the hands from the arms. However, for this method, the face should be in the image, and its use gets limited when there are multiple people. In order to divide an arm region, Malassiotis and Strintzis [11] sequentially scan the depth image, perform the initial clustering, and divide each pixel from the initially clustered pixels based on the distance from its neighbors. Then, the neighboring clusters are combined, and the arm region is finally detected. The arm's coordinates are statically modeled in a 3D space to divide the hand from the forearm. The Gaussian mixture model is used for calculating the probability distribution of the 3D *x*-coordinates and then to detect the hand and the forearm regions. This method detects a static pose, but it is limited when used for dynamic gesture recognition because the distribution model needs to be revised when the depth data change. Suryanarayan et al. [12] proposed 2D figure data, a compressed 3D figure descriptor, and a 3D volume metric figure descriptor for hand pose recognition that uses depth data. The hand is detected by creating a histogram of depth values and the detected hand is separated from others by Otsu's threshold method. This method is also limited when there is another object between the camera and the hand. Oikonomidis et al. [13] used a Kinect camera sensor [14] to detect a hand. It uses the hand model with all degrees of freedom. Then, it initializes the hand model with the hypothesized pose and keeps tracking a hand in real time by updating the hand model. This method optimizes the hand model parameters through minimizing the difference between the assumed hand model in the 3D space and the actual hand. However, it recognizes the hand pose by comparing adjacent distances; therefore, an error may occur because the hand pose becomes increasingly blurry with an increase in the distance.

**Figure 1:**
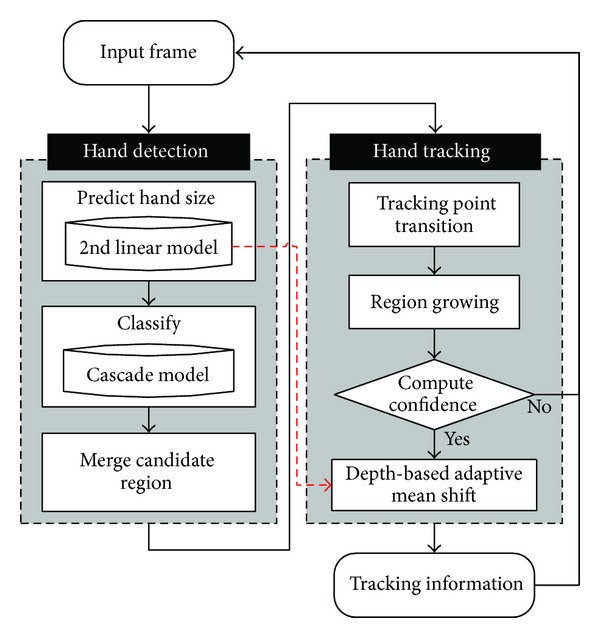
System flow.

Figure 1 shows system flowchart. The proposed system consists of main two steps with hand detection and tracking. In this study, we use the depth data inputted from the Kinect camera sensor. We suggest the detection and tracking method for stable hand detection irrespective of the experiment lighting conditions and color data. Further, we quantitatively and qualitatively analyzed the results of the hand region tracking per distance from the camera and per speed. We evaluate the effectiveness of the system by examining the mean hand detection speed, computing speed, and tracking performance as well.

The rest of this paper is organized as follows: Section 2 explains the features for real-time hand detection. It also describes the learning and recognition processes.  Section 3 explains the tracking process introducing Depth-based Adaptive Mean Shift (DAM-Shift) algorithm. DAM-Shift algorithm refers to the hand's center point acquired from the previous step when it begins its operation in the current frame.  Section 4 describes a testing environment and the experimental results to prove the effectiveness of the proposed algorithm. Lastly, in Section 5, we present the conclusions and a suggestion.

## 2. Hand Detection

### 2.1. Dynamic Depth-Based Difference Feature

The purpose of features is to ease the fast and accurate detection of the hand region in a depth image [15]. We use very simple features and let the boosting and cascade method learn how to detect a hand region using the features. AdaBoost is popular for object detection, especially face detection. Viola and Jones [16] used the Haar-like features to detect a face and achieved good results because a face has certain distinct characteristics. However, a hand does not contain distinct characteristics, and a depth image would have only the shape information. The use of shape information requires a segmentation of an object, which is a very complex process. We suggest simple though very effective features to detect multiple hand regions.

Algorithm 1 shows the proposed algorithm for feature extraction. When an image segment *I* and control parameters *N*_*x*_ and *N*_*y*_ are inputted, *I* is divided into blocks whose width and height are block_*w*_ and block_*h*_, respectively. step_*x*_ and step_*y*_ denote the amount displacement of included blocks along an *x*-axis and *y*-axis, respectively. End_*x*_ and End_*y*_ represent the number of blocks along a horizontal and vertical direction. The total number of included blocks becomes End_*x*_ × End_*y*_.

**Figure 2:**
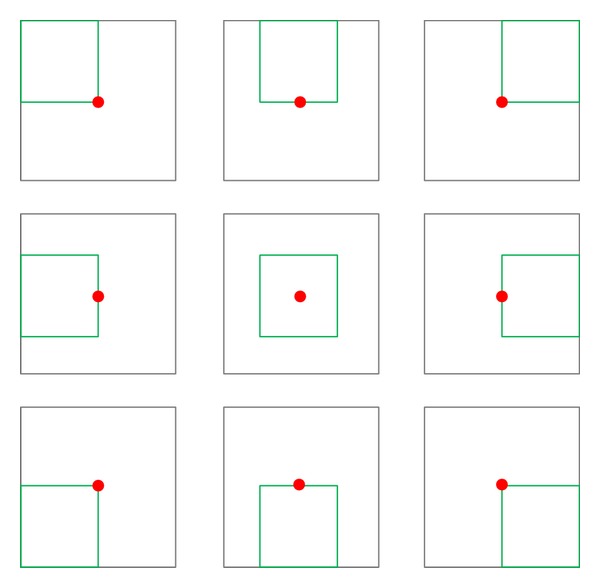
Example of extracting features.

Figure 2 illustrates how features are extracted with an example where *N*_*x*_ = 2 and *N*_*y*_ = 2. The inside quadrangles represent included blocks. Since *N*_*x*_ = 2 and *N*_*y*_ = 2, the number of included blocks is End_*x*_ × End_*y*_ = 9. A feature value is computed at each block. An average depth value of each block is subtracted from the central depth value at the position of the center dot, and the resulting value is used as the feature. The features would be used for learning the classifier. To find the best features, the learning process examines goodness of features varying the control parameters; *N*_*x*_ = {1,…, *n*}, *N*_*y*_ = {1,…, *m*}.

The function area of Algorithm 1 returns the sum of depth values in the rectangle roi, which requires a substantial amount of computing time. We effectively resolve this problem using an integral image [16]. The average depth of roi is subtracted from the central depth value Depth_*c*_, and the resulting value is stored in an array Fv as a feature value.

### 2.2. Learning and Classification

The computing time for the feature values can be reduced using an integral image. A cascade method can further reduce the computing volume for positive and negative decisions, enabling real-time detection. However, scale invariance is another issue to be resolved for object detection. For example, in [16], the AdaBoost predetermines the size of a face or it scans all the possible sizes for face detection. We solve this problem by predicting the size of a hand. The prediction is made using the depth value of the hand region. It enables detecting all sizes of hand regions within a single scan. Even though the size of a hand differs individually, we assume that this variance is insignificant. Therefore, we predict the size via the 2nd polynomial model as follows [17]:

(1) $$\begin{array}{c} r=\left[\begin{array}{ccc} 1 & x & x^{2} \end{array}\right]\left[\begin{array}{c} \alpha_{1}\\ \alpha_{2}\\ \alpha_{3} \end{array}\right], \end{array}$$

(2) $$\begin{array}{c} \alpha ={\left(P^{T}P\right)}^{-1}P^{T}y, \end{array}$$

(3) P=[1x1x121x2x22⋮⋮⋮1xnxn2],  y=[r1r2⋮rn].

Equation (1) is to estimate the radius of the enclosing circle of a hand region using the 2nd polynomial model. The value of *x* in (1) is the representative depth of a candidate hand region in the current frame. To obtain the value *r*, we need the coefficients $\alpha =\left[\begin{array}{ccc} \alpha_{1} & \alpha_{2} & \alpha_{3} \end{array}\right]$. These coefficients can be learned using (2) and (3). The values of $\left(\begin{array}{cc} x_{i} & r_{i} \end{array}\right)$ are collected manually at the learning phase. We measure the size of a hand region varying the depth of a hand to different values. We have made an assumption that the size of hand can be represented as the 2nd polynomial function of a depth. Thus, once *α* is determined through learning data, the size of the hand region can be estimated using (1).

**Figure 3:**
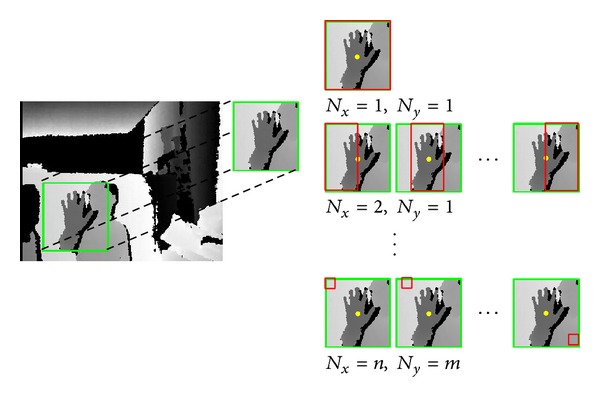
Example of feature extraction.

A learning phase needs features to be used when building a boosting classifier. The pool of features is made with an algorithm illustrated in Algorithm 1 by varying the control parameters *N*_*x*_ and *N*_*y*_.  Figure 3 shows an example of building a pool of features. Given *n* and *m*, features are extracted from the given region *N*_*x*_ = 1, *N*_*y*_ = 1 until the condition *N*_*x*_ = *n*, *N*_*x*_ = *m* is met. Hence, the total number of features in a pool becomes as in

(4) $$\begin{array}{c} N_{\text{feature}}=\overset{m}{\underset{N_{y}=1}{\sum }}\overset{n}{\underset{N_{x}=1}{\sum }}\left(2N_{x}-1\right)\left(2N_{y}-1\right). \end{array}$$

The values of *n* and *m* are to be determined according to the size of a hand region. For example, when the size of a hand region is around 20 by 20, *n* and *m* can be set to 10. For *n* = *m* = 10, the total number of features becomes 10000.

When a pool of features is provided, a learning process can operate through a boosting classifier. Most classifiers try to increase a detection rate (number of detected positive samples over total number of positive samples), while they try to reduce a false positive rate (number of detected negative samples over total number of negative samples). In general, these two criteria contradict. The AdaBoost algorithm proposed by Viola et al. determines the threshold value with the least error rate to form a weak classifier. Then, a strong classifier combines weak classifier so that it could meet some desired detection rate. If the strong classifier could not meet satisfactory false positive rate, the next stage of the cascade structure is carried out. That is, each stage of the cascade structure works as a strong classifier. It results in that a very large number of weak classifiers are needed in the overall cascade structure.

Because a hand region is rather regular and has a simple pattern, we rather choose a weak classifier that acts like a strong classifier of Viola. A single weak classifier is to perform the role of a strong classifier of Viola by setting its threshold value so that it could meet a desired detection rate. In other words, we enforce a kind of overfitting at each weak classifier. A satisfactory false rate is then handled in the succeeding stages of a cascaded structure in a sequential manner. This strategy could speed up the detection procedure and reduce the number of weak classifiers dramatically:

(5) $$\begin{array}{c} d_{r}=\max\left(\frac{\left(T^{+}-S^{+}\right)}{T^{+}},\frac{S^{+}}{T^{+}}\right), \end{array}$$

(6) $$\begin{array}{c} e_{r}=\min\left(S^{+}+\left(T^{-}-S^{-}\right),S^{-}+\left(T^{+}-S^{+}\right)\right). \end{array}$$

**Figure 4:**
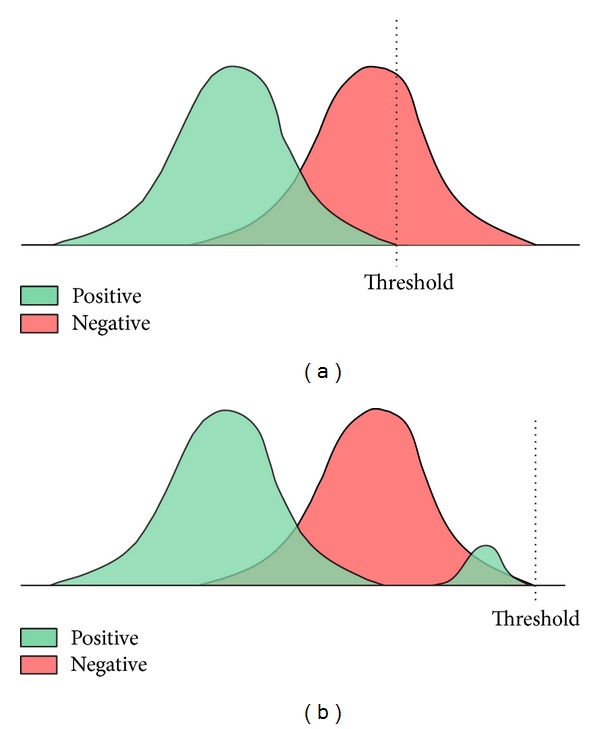
Method for calculating the threshold.

Equations (5) and (6) are to compute the detection rate and error rate of a weak classifier, respectively. *S* ^+^ and *S* ^−^ denote the weighted sums of positive and negative samples below and above the threshold value, while *T* ^+^ and *T* ^−^ denote the total weighted sums of the positive and the negative samples. That is, referring to Figure 4, *S* ^+^corresponds to the positive sample area below the threshold value and *S* ^−^ corresponds to the positive sample area above the threshold value, while *T* ^+^ corresponds to the overall positive sample area and *T* ^−^corresponds to the overall negative sample area.

**Figure 5:**
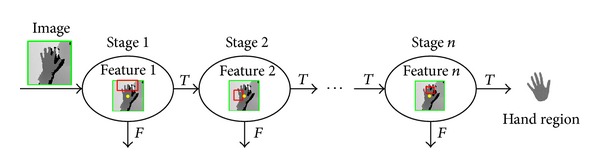
Structure of classifier generated using cascade.

Figure 4 shows the position of the threshold value where the error rate becomes minimum while keeping the maximum detection rate for a given feature. In Figure 4(a), the given threshold value allows the maximum detection rate as well as the minimum error rate. In Figure 4(b), the given threshold value gives the maximum detection rate, though it causes a very big error rate. In practice, the threshold value gets adjusted to decrease the error rate until it meets the desired detection rate. Each stage of a cascade structure, as in Figure 5, would be constructed as a single weak classifier maintaining the desired detection rate and sequentially reducing the false positive rate.

Figure 5 shows the cascaded structure of the proposed classifier. The appearance of a hand region is restricted to an open palm so that the shapes of all possible hand regions remain similar while only their sizes differ. Each stage chooses the best feature to form a classifier. The threshold value of the chosen feature is determined in order to minimize the false positive rate while maintaining the desired detection rate. The classifier of each stage accepts most of positive samples while rejecting as many negative samples as possible. Therefore, the desired detection rate is maintained even after it passes several stages, and the false positive rate gets reduced as the number of stages increases. For example, if we set the required false positive rate of each stage to be 0.7, the overall false positive rate becomes 0.000798  (0.7^20^) after 20 stages pass.

Algorithm 2 presents a simple form of the classifier building algorithm used in the current study. *F*_*i*_ denotes the current false positive rate, and *F*_desired_ and *D*_desired_ represent the desired false positive and detection rates, respectively, which are the constants that a user selects in advance. *N* denotes a set of training image segments. The algorithm shows that, out of features that satisfy the aforementioned threshold determination rule, a feature that satisfies *d*_*r*_ ≥ *D*_desired_ and has the lowest *e*_*r*_ value is selected. When *D*_desired_ is near 1, *e*_*r*_ could be more than 0.5. In order to solve this problem, if the value of *e*_*r*_ is more than 0.5 in the next step, the algorithm reduces *D*_desired_ and repeats the process of selecting the best feature.

Once a feature is selected, the cascade classifier, a combination of the selected feature and the previously selected features, computes *F*_*i*_. A weak classifier with the selected feature is generated, and it becomes the component of a cascade. In the next stage, the cascade classifier organizes a set of learning samples *N* only with all of the positive samples and falsely detected negative samples and repeats the process until *F*_*i*_ becomes smaller than *F*_desired_.

**Figure 6:**
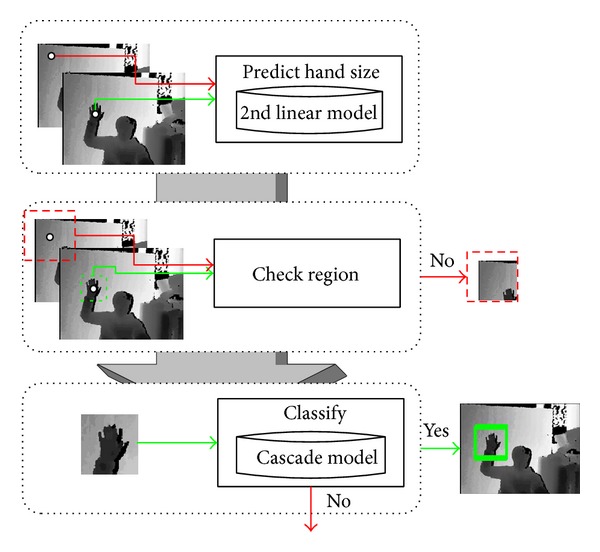
Process of hand region detection.

Figure 6 shows the process of hand region detection using the 2nd polynomial model and the generated cascade classifier. The detection process consists of two steps. For each candidate pixel position of a hand region, the prelearned 2nd polynomial model predicts the size of a hand region. Based on the size, a rectangular region is generated with the center at the candidate pixel position. If the generated region is completely inside an image, the cascade classifier determines whether the generated region is a hand region or not. When multiple pixels are classified as centers of hand regions (i.e., multiple regions are found as hand regions), they are tested whether they can be combined to form one hand region through the merge operation.

Algorithm 3 shows the merge algorithm. In Algorithm 3, distance(*A*, *B*) represents the depth difference between the center points of Quadrangle *A* and Quadrangle *B*, and intersect(*A*, *B*) denotes the size of the overlapped area of Quadrangle *A* and Quadrangle *B*. Further, Th_*d*_ denotes the threshold value of the depth difference, and Th_*r*_ represents the threshold value of the overlapped region. In the test, we used the values of 50 and 0.6, respectively.

TempR denotes the memory space where the data for many quadrangles are stored. In other words, we test the depth differences and the overlap regions between the center points of the quadrangles, which are hand region candidates, and any quadrangle that passes the test is added to the memory space TempR. When another quadrangle is tested, the depth difference and the degree of overlap with the average quadrangle in TempR are compared for the merge operation. The final product of the merge process is the average quadrangle, which becomes the final hand region. The average quadrangle is found by the average coordinate of the vertex of each quadrangle. Furthermore, in the next tracking stage, the center point of the average quadrangle is set to the initial point of the tracking.

**Figure 7:**
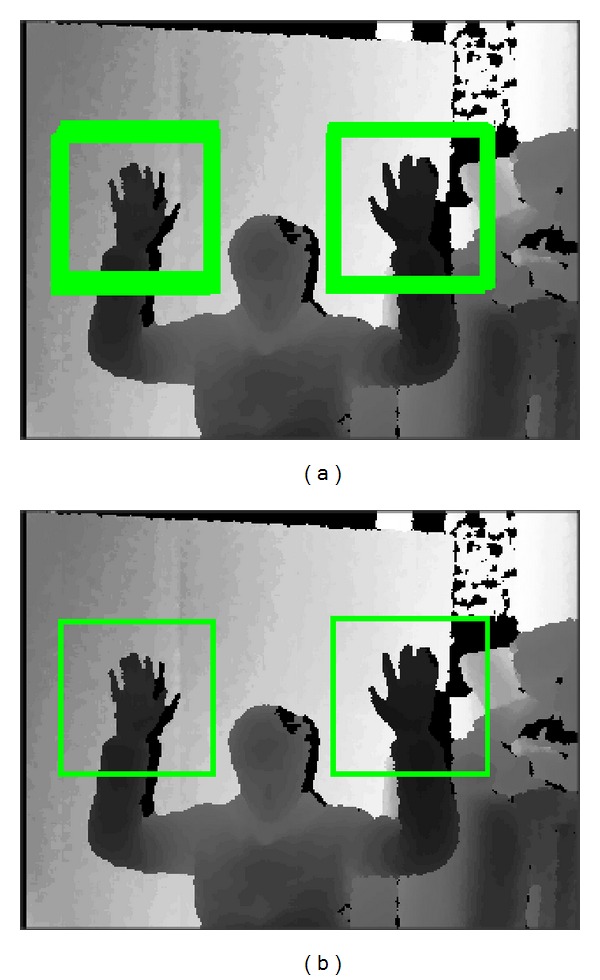
Result of merge process.

Figure 7(a) shows an image generated after all the pixels are classified, and Figure 7(b) shows the final result obtained by executing the merge algorithm.

## 3. Hand Tracking

### 3.1. Tracking Point Transition and Region Growth

In the previous section, the hand region and the center point of the hand region are detected. In the tracking process, the nearest point in the next frame is to be detected on the basis of the center point of the hand region in the current frame [18]. The nearest point becomes the base for the tracking using the region growth method and the depth-based adaptive mean shift algorithm.

**Figure 8:**
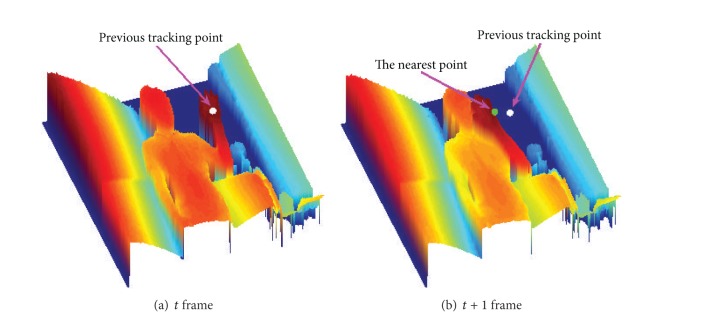
Transition of tracking point.

Figure 8 shows an example of the tracking point transition. First, (a) shows a depth image including the center point of the hand region found in the detection stage, and (b) illustrates the next frame and shows the changed position of the hand. We suggest a very simple yet effective method to track a hand. As shown in (b), the nearest point is found from the previous tracking point.

The term nearest point means the closest point in (*x*, *y*, *z*) space. In (7), TP_*t*_^pre^ denotes the tracking point of the previous frame and *p* represents a random point for the sake of comparison. Equation (8) is used for calculating the Euclidean distance. While the distance in *x*- and *y*-axis is merely the coordinate difference between the two points, the difference in *x*-, *y*-, and *z*-axis (the depth), is calculated in (9). In (9), *T*_*f*_ denotes a constant, which is the weighted value to the nearest point from the camera, because the hand region lies in front of the other body parts when, in general, one makes a gesture. Further, *T*_dc_ compensates the difference in the units of axis *z* and axes *x* and *y*. We have used the values of *T*_*f*_ = 50 and *T*_dc_ = 4 on the basis of our experiences from previous experiments:

(7) $$\begin{array}{c} \text{T}{\text{P}}_{t}^{\text{seed}}=\text{argmin}\left(d\left(p,\text{T}{\text{P}}_{t}^{\text{pre}}\right)\right), \end{array}$$

(8) $$\begin{array}{c} d\left(a,b\right)=\sqrt{{\left(a_{x}-b_{x}\right)}^{2}+{\left(a_{y}-b_{y}\right)}^{2}+zd\left(a_{z},b_{z}\right)}, \end{array}$$

(9) $$\begin{array}{c} zd\left(a,b\right)={\left(\frac{\left|a-\left(b-T_{f}\right)\right|}{T_{\text{dc}}}\right)}^{2}. \end{array}$$

When the nearest point TP_*t*_^seed^ is found from the tracking point of the previous frame, a hand region can be inferred using the point as a seed. We use the region growing method to detect a hand region from the nearest point. The depth value is fundamental in the region growing method. That is, if the depth value is similar, the region growing can carry on. However, the grown region could be considerably broad if the depth value of an object changes gradually. Therefore, the global threshold value is used to avoid this problem:

(10) $$\begin{array}{c} {\text{Cond}}_{\text{all}}=\{\begin{array}{cc} \overset{n}{\underset{i}{\sum }}\;{\text{cond}}_{i}\geq n & \text{True}\\ \text{otherwise} & \text{False}, \end{array} \end{array}$$

(11) $$\begin{array}{c} {\text{Cond}}_{1}=\{\begin{array}{cc} \left|D_{t}\left(n,m\right)-D_{t-1}\left(c,r\right)\right|<{\text{Th}}_{D} & 1\\ \text{otherwise} & 0, \end{array} \end{array}$$

(12) $$\begin{array}{c} {\text{Cond}}_{2}=\{\begin{array}{cc} D_{t}\left(n,m\right)-{\text{Dep}}_{\text{TP}}<{\text{Th}}_{\text{GD}} & 1\\ \text{otherwise} & 0, \end{array} \end{array}$$

(13) $$\begin{array}{c} {\text{Cond}}_{3}=\{\begin{array}{cc} {\text{Grow}}_{\text{cnt}}<{\text{Th}}_{c} & 1\\ \text{otherwise} & 0, \end{array} \end{array}$$

(14) $$\begin{array}{c} {\text{Cond}}_{4}=\{\begin{array}{cc} \text{Dist}<{\text{Th}}_{c} & 1\\ \text{otherwise} & 0. \end{array} \end{array}$$

Equations (10)–(14) specify conditions where the region growing would be conducted. In (11), *D*_*t*_(*n*, *m*) denotes the depth value of the pixel for which the region-growing test is to be performed and *D*_*t*−1_(*c*, *r*) denotes the depth value of the base pixel where the region growing was performed in the previous iteration. That is, when compared to the depth value of the base coordinate, if the difference of the depth value is below the threshold Th_*D*_, it satisfies the condition. Equation (12) expresses the condition that limits the range of the region growing. Dep_TP_ denotes the depth value of TP_*t*_^seed^, so that it limits the depth value range on the basis of the global threshold Th_GD_. Grow_cnt_ in (13) accumulates the amount of region growing in order to limit the size of the region. Dist in (14) denotes the Euclidean distance between the nearest point TP_*t*_^seed^ and the pixels that is under consideration for region growing. When all conditions, (11)–(14), are met according to (10), the value becomes true and the corresponding pixel is included in a hand region.

Algorithm 4 presents the region growing algorithm. The threshold values of depth threshold Th_*D*_ and the depth threshold of global Th_GD_ are inputted, and the nearest point TP_*t*_^seed^ is calculated using (7). Dep_TP_ means the depth value of TP_*t*_^seed^. Once Dep_TP_ is inputted, the threshold value of Th_*c*_ can be obtained from the previously generated 2nd polynomial model. Initially, the coordinate array *B* is filled with the base coordinate TP_*t*_^seed^. We examine eight neighbors of the newly incoming coordinates of *B* to see whether the conditions in (10) are met and include in the array *B* the coordinates of the neighbor which meets the conditions. The process of region growing is repeated for appropriate pixels. When these conditions Cond_all_ are not met, the failed pixels are stored in two ways. If Cond_1_ is not met, the pixels are added to the set of valid borderlines VB, whereas the others are added to the set of invalid borderlines UVB. The set of valid borderlines, which is a result of region growing, is later used in the DAM-Shift algorithm.

**Figure 9:**
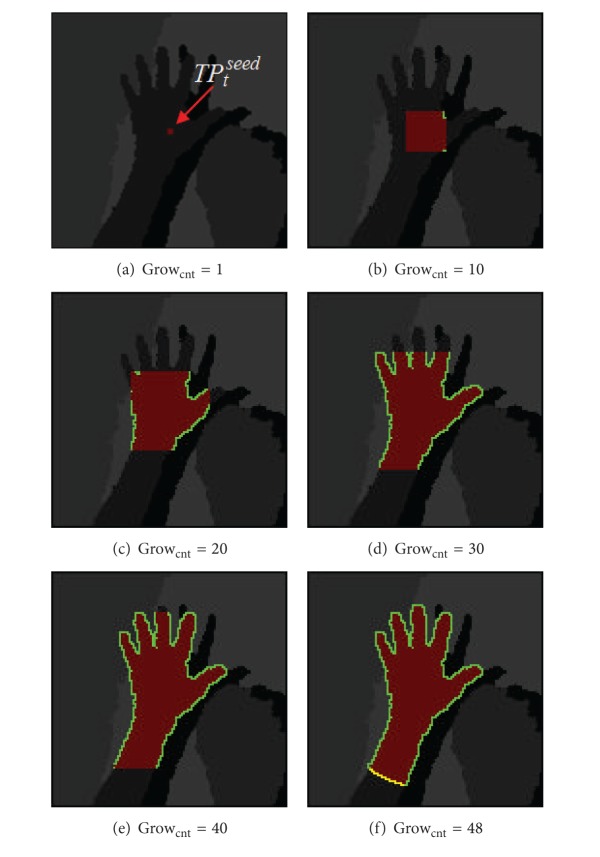
Result of region growth.

Figure 9 shows the result of implementing the algorithm presented in Algorithm 4. The conditions given in (10) are tested, and the region grows repeatedly as shown in the figure. Th_*c*_ is evaluated to be 48, given the depth value of TP_*t*_^seed^. This implies that the selections are repeated 48 times to complete the region growing.  Figure 9(f) shows green and yellow borderlines for the pixels that did not meet Cond_all_. The green line denotes a valid borderline VB, and the yellow line represents an invalid borderline UVB.

### 3.2. Depth-Based Adaptive Mean Shift (DAM-Shift)

In the tracking process, the nearest point is detected from the previous tracking point, the region is grown, and the hand region is detected. For stable tracking, a point that meets certain conditions needs to be tracked. Hence, we have defined a point that converges to the center of the contour line using the DAM-Shift algorithm as the tracking point. The DAM-Shift is defined in a manner similar to the Mean Shift [19], but its kernel size adaptively changes according to the depth values and the iteration time:

(15) $$\begin{array}{c} \text{T}{\text{P}}_{i+1}^{\text{mean}}=\frac{\sum_{p\in \Omega }p\cdot K\left(p,\text{T}{\text{P}}_{i}^{\text{mean}},\text{DT}{\text{P}}_{t-1}^{\text{mean}},i\right)}{\sum_{p\in \Omega }K\left(p,\text{T}{\text{P}}_{i}^{\text{mean}},\text{DT}{\text{P}}_{t-1}^{\text{mean}},i\right)}, \end{array}$$

(16) $$\begin{array}{c} K\left(p,s,d,i\right)=\{\begin{array}{cc} 1 & ||p-s||<R\left(d,i\right)\\ 0 & \text{otherwise}, \end{array} \end{array}$$

(17) R(d,i)={SPM(d)2SPM(d)−i·Trc<SPM(d)2SPM(d)−i·Trcotherwise,

(18) $$\begin{array}{c} \text{SPM}\left(d\right)=\alpha_{1}+\alpha_{2}d+\alpha_{3}d^{2}. \end{array}$$

Equation (15) shows how the DAM-Shift algorithm works. Here, *K*(·) denotes a kernel function whose size adaptively varies according to the depth of the tracking point. TP_*i*+1_^mean^ depicts the coordinates of the tracking point at the *i* + 1 iteration. It is updated as the iteration goes on. DTP_*t*−1_^mean^ represents the depth value of the tracking point of the previous frame. *p* denotes a point that belongs to the set *Ω*, where *Ω* represents the set of valid borderlines VB that is acquired during the region growing. The coordinates of the nearest point TP_*t*_^seed^ is obtained as in Section 3.1, and it is substituted for TP_0_^mean^. As the iteration goes on, TP_*i*+1_^mean^ is replaced with TP_*i*_^mean^ for the next iteration. The process is repeated until the point of convergence. The kernel function's radius *R*(*d*, *i*) changes according to the depth value and the number of repetition. Equation (17) is to calculate the radius depending on depth and repetition. For this purpose, the function SPM(*d*) is used, which corresponds to the 2nd polynomial model defined in Section 2.2. Beginning with the double size of SPM(*d*), the radius keeps on decreasing as the iteration goes on. But, to avoid an infinite decreasing, the base radius SPM(*d*) is used if the considered radius is smaller than the base value. We have defined the radius-reducing constant *T*_rc_ on the basis of our empirical knowledge.

An image of the hand region with the five fingers open is acquired from various distances, and the center point of the palm is determined manually. The distance from the center point to the farthest middle finger and the depth value of the central palm are collected. The depth value is assigned to *x* in (3), where *r* is set to be the distance from the center point to the farthest middle finger. Then, (2) is used for calculating the values of *α*_1_, *α*_2_, and *α*_3_ to be used in (18).

**Figure 10:**
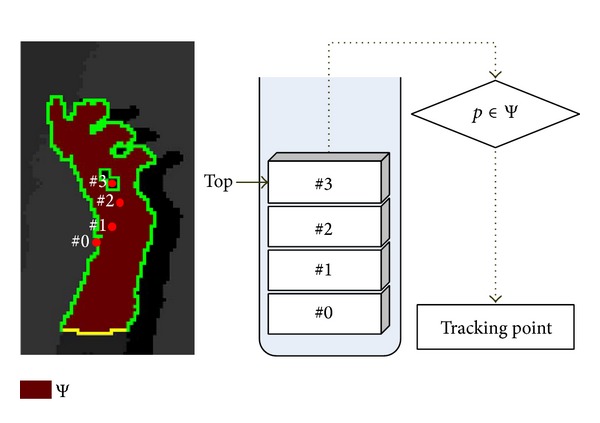
Decision process for tracking point.

Figure 10 shows the process of determining the tracking point through the DAM-Shift algorithm. The DAM-Shift does not guarantee a smooth tracking. The converged point may not fall in the hand region Ψ, though it rarely happens. It is because only border pixels are involved in the process of determining a tracking point. To fix this problem, we use a stack that keeps the history of convergence of a tracking point. In Figure 10, the left figure shows the result after the DAM-Shift algorithm is executed through the kernel function. Number 0 denotes the initial point, and its depth value is assigned to the kernel function to get the next point number 1. Suppose the final converged point number 3 is located outside the hand region (number 3 ∉ Ψ). It may lead to an error when determining a tracking point in the next frame. As shown in the right figure of Figure 10, we store the resulting coordinate of each run of the DAM-Shift algorithm in the stack-structured memory. Once the DAM-Shift algorithm converges, a pop operation is executed. The top coordinate of the stack is checked to see whether it is inside the hand region or not. The first successful coordinate becomes the final tracking point.

### 3.3. Detection of Inappropriate Tracking

While tracking, many unexpected things may happen. A user may want to finish a hand movement or a hand may touch other objects. To handle situations where tacking is impossible or unnecessary, we need to judge the success or failure of tracking, whenever the region growing is completed. After many experiments, we have found that the tracking point moves inappropriately when a hand moves very swiftly, or hand is positioned behind the face or overlapped with another object. Hence, we include a module that detects such invalid tracking cases.

**Figure 11:**
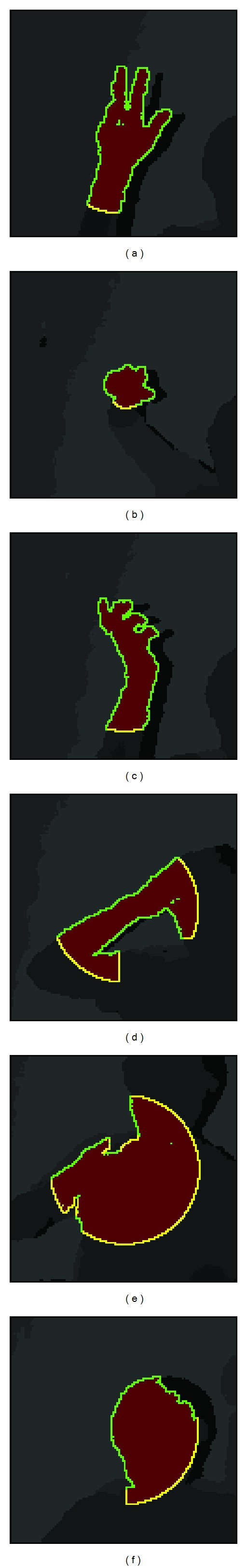
Results of region growing and boundary detection.

As shown in Figure 11(a) through Figure 11(c), the invalid borderline (the yellow one) is, in general, very short when the hand region is normally detected. In contrast, when the hand region overlaps with another region as shown in Figures 11(d) and 11(e) and when the hand region detection moves to the face region after its failure as shown in Figure 11(f), the length of invalid borderlines is considerably longer than that of the invalid borderlines of a normal hand region. Therefore, this observation is used for judging whether the hand region is being tracked normally.

Equation (19) computes a reliable threshold value using a formula to calculate the circumference. Th_*c*_ denotes the threshold value used in (13) and (14), and it changes adaptively according to the depth value of the nearest point. A value of 0.35 is determined empirically. If the number of pixels in the invalid borderline is less than Th_confidence_, the tracking is considered successful, while the opposite is considered a failure. During region growing, borderline detection is performed and simultaneously a test is made to judge the success or failure of the tracking by finding the valid and invalid borderlines:

(19) $$\begin{array}{c} \text{T}{\text{h}}_{\text{confidence}}=2\pi \times \text{T}{\text{h}}_{c}\times 0.35. \end{array}$$

## 4. Experimental Results

We used depth images of 320∗240 created by Microsoft Kinect as an input device. The above mentioned algorithms, hand region detection and a tracking, are tested with Intel(R) Core(TM) Quad CPU 2.66 GHz and 3-GB memory.

**Figure 12:**
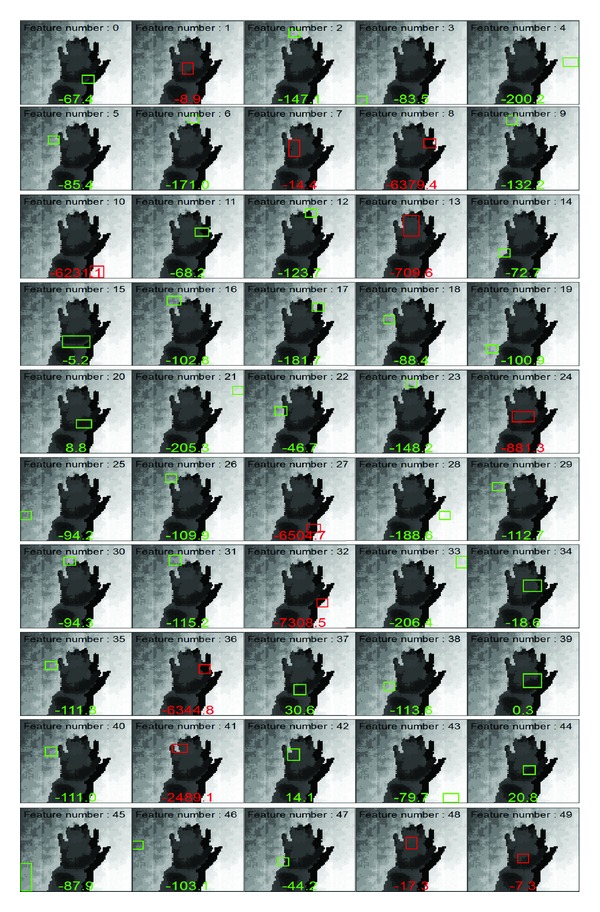
Fifty selected features.

Figure 12 shows 50 features that were selected during the learning, out of 10,000 possible features. The number at the bottom of each image is the threshold value of the feature, and the green quadrangle represents Parity = 1, whereas the red quadrangle denotes Parity = −1. Parity = 1 implies that the difference between the depth of center and the average depth of quadrangle region is smaller than the given threshold, while Parity = −1 implies the reverse way. That is, the green quadrangle region is farther away from a camera than the central pixel. Most of the red quadrangles are located within the hand region, which implies that the difference between the center and the average quadrangle region is larger than the threshold. In the case of number 10 shown in Figure 12, the red quadrangle is located outside a hand region, but it works for detection because of the small threshold value, −6231.1. Further, the collection of 50 features looks similar to the shape of a hand; hence, the purpose of feature selection is intuitively understood.

Table 1 summarizes experimental results, comparing our method against Discrete AdaBoost, Real AdaBoost, and Gentle AdaBoost [20]. The tests have been carried out using the same data set to all the methods. Discrete AdaBoost, Real AdaBoost, and Gentle AdaBoost methods require the maximum allowable false positive rate as an input parameter for each stage when they build a classifier. It is set to vary from 0.7 to 0. On the other hand, our method does not need the range of false positive rates to complete each stage. DR denotes the detection rate, and FDR refers to the false discovery rate. Computation denotes a computing time (ms) until a detection process is completed for each frame.

In general, our method surpasses the other boosting methods in every criterion. Gentle AdaBoost exhibits the best result where the stage-allowed MFPR is set to zero. As shown in the table, the detection rate in this case is 0.98, and the false discovery rate is 0.01. However, the detection speed is twenty times slower than that of our method. This was attributed to the fact that the stage has to have all the possible weak classifiers since MFPR was set to zero. The increase in the number of weak classifiers results in the decrease in the speed. Our method does not select any incorrect hand image at a detection rate of 97% and has the fastest computation speed compared to other algorithms. This is because our classifier contains only 50 features and forms a cascading structure.

**Figure 13:**
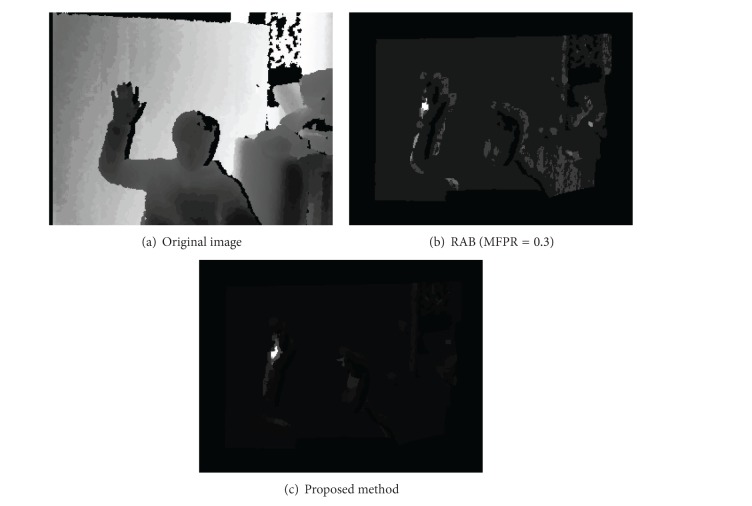
Computational performances.

Figure 13 shows the classifier's computational performances. (a) shows the original image, (b) shows the result of a combination of Real AdaBoost and Cascade [20], and (c) shows the result of the proposed method. For the black regions in (b) and (c), a classification is not performed because they did not meet the region test conditions illustrated in Figure 6. The white pixels represent positions which are classified as hand region positions. The pixel values of the remaining region imply the number of classifier where they are evaluated during the classification process. In other words, the darker pixels are claimed as nonhand region position within a fewer number of classifier. As seen in the images, the white pixel positions illustrated in (c) show the presence of a hand region more accurately than in (b), and the remaining pixels in (c) are considerably darker than those in (b). Therefore, the proposed method is better in terms of speed and accuracy.

**Figure 14:**
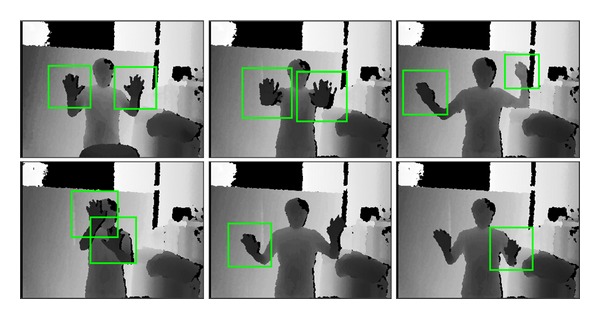
Detection results.

Figure 14 shows depth images with detected hand regions in quadrangles. The green quadrangles are the ones obtained after the detected quadrangles are merged. Most of the hand regions are accurately extracted. The hand regions used in a learning phase are front-faced open palms. Because the features refer to the difference between the depth of center and the average depth of a region, rather than the details of the hand figure, slightly deformed hand regions are extracted. This could be improved by an addition of more positive samples. Many tests prove that the FDR is considerably low.

**Figure 15:**
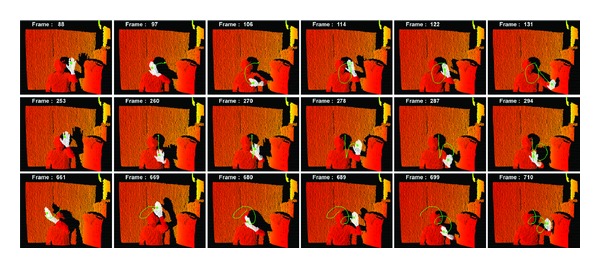
Tracking results.

Table 2 presents the measured values of the tracking accuracy of the hand region. As shown in Figure 15, while a hand draws “a,” “b,” and a spiral, the trajectory of its movement is stored, and the average error of the trajectory is evaluated by changing the distances from a camera. The error is calculated in differences of pixel positions between ground truth points and the tracking points. Ten tests are performed, and their results are averaged. The closer a hand is, the greater the error occurs because a closer hand gives more displacement and its hand region is bigger even at the same speed than a far way hand. However, most of the results are within 5 pixels, indicating a good performance.

Figure 15 shows the tracking data of Table 2 and the trajectory of the hand region tracking. The green point denotes the tracking point where the green line is a sequence of tracking points. The number on the upper left of each image is the frame number. The first row of the images shows a drawing of “a,” the second row illustrates a drawing of “b,” and the last row shows a drawing of a spiral. Each drawing is completed in 1.5 sec.

**Figure 16:**
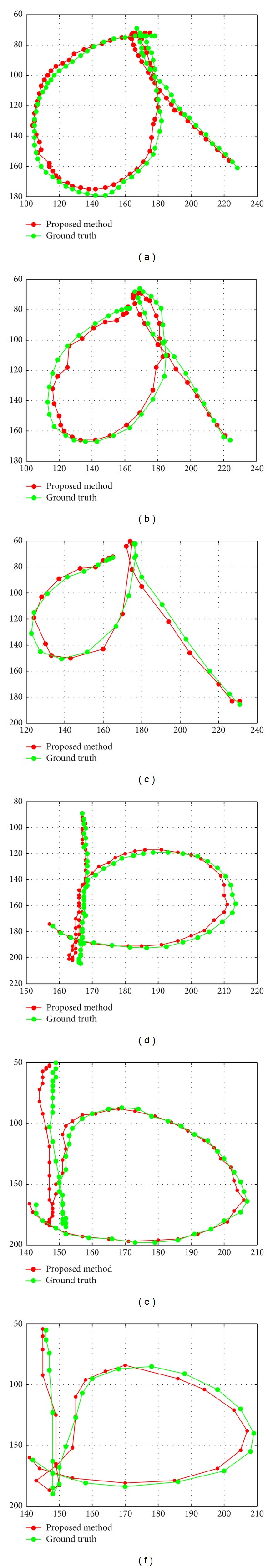
Tracking results of gestures “a” and “b” under various velocity conditions.

Figure 16 shows the 2D pictures of tracking paths of letters “a” and “b” after a hand region is detected from a distance of 1.2 m from a camera. (a) and (d) on the left show slow speed, whereas (b) and (e) in the middle illustrate normal speed, and (c) and (f) on the right show the fast speed. The red paths are ones obtained by using the proposed method, and the green paths represent the ground truth of the center points of the hand obtained manually. In most cases, the tracked paths shown in the figures are free of errors with respect to the ground truth.

Table 3 presents the average tracking errors compared with those of the ground truth after ten gestures of “a” and “b” at each speed from 1.2 m distance. Most of the gestures have errors of less than five pixels. In the case of “a,” at a fast speed, more errors occurred as compared to in the case of the other gestures. It is because the last stroke of a letter “a” is completed considerably fast due to the feature of the letter. Hence, the depth image of the hand region becomes blurred, which results in an inaccurate tracking of the center point of a hand.

**Figure 17:**
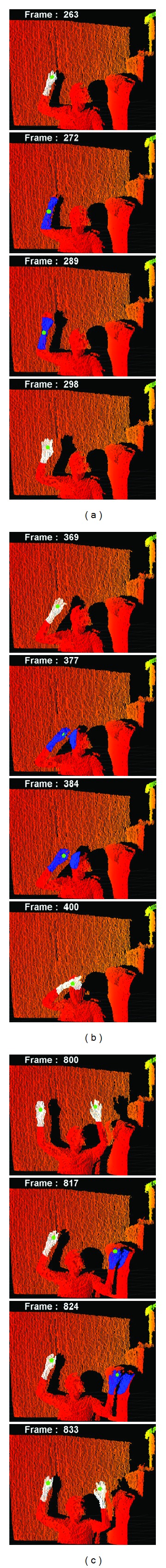
Example of tracking confidence.

Figure 17 shows results of invalid tracking that occurs during judging an inappropriate tracking explained in Section 3.3. The white regions denote hand regions that are normally tracked, while blue regions represent hand regions that do not pass the reliability test and are hence considered to be failed hand regions. In (a), a hand region could not pass the reliability test as a hand is close to a wall. In (b), a hand touches a face and is then detached from it. Further, in (c), a right hand touches a neighbor object and is then detached from it. We have let the tracking continue for a certain length of time even though the region does not pass the reliability test. Thus, any tracking that becomes normal within a certain time limit is allowed to continue. Hence, all the images at the bottom of the figure show normal tracking.

Table 4 shows average computing times for the detection and tracking processes. In the case of an irregular gesture, we have let the detection and tracking processes run ten times for 3 min. The average computing times are evaluated. In the detection stage, the 2nd polynomial model detects a hand region in a single scan when the pixels are fed. Its computing speed is excellent. The tracking stage for the nearest point detection, the region growing, and the DAM-Shift is considerably fast because of a low computing volume.

## 5. Conclusions

We have proposed the hand detection and tracking method that works very well in a real world environment. For hand detection, we have developed very effective features and the cascade structure of a classifier. The features are generated based on dynamic depth differences. The cascade structure is constructed with selective employment of features according to their discriminating power with the strategy of minimizing a false positive rate at each stage.

For tracking a hand, we have developed DAM-Shift algorithm which is a variant of CAM-Shift algorithm. DAM-Shift algorithm varies its search area according to the depth of a hand. Our 2nd polynomial model works well to predict the size of a hand, which plays an important role in confining a search area. To handle situations where tacking is impossible or unnecessary, we have developed the judgment module which detects an inappropriate tracking. The judgment module can decide whether current tracking is valid or not.

We have evaluated the proposed hand detection and tracking algorithm by comparing it against the existing AdaBoost algorithms qualitatively and quantitatively. We have analyzed the tracking accuracy through performance tests in various situations. Current study shows that the proposed methods surpass the existing other methods in terms of accuracy and computation time.

## Figures and Tables

**Algorithm 1:**
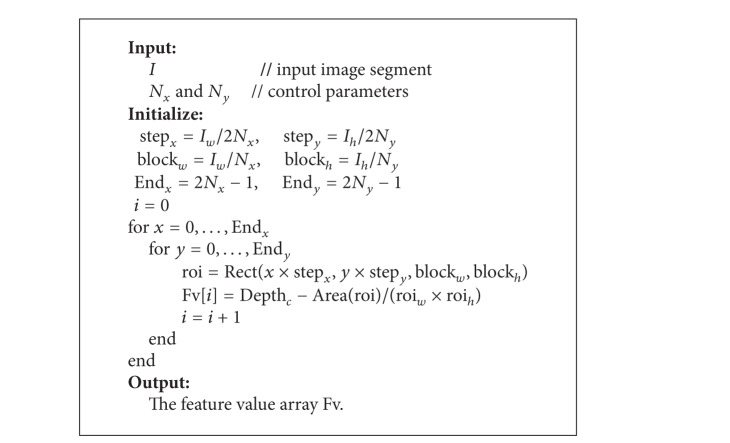
Proposed algorithm for feature extraction.

**Algorithm 2:**
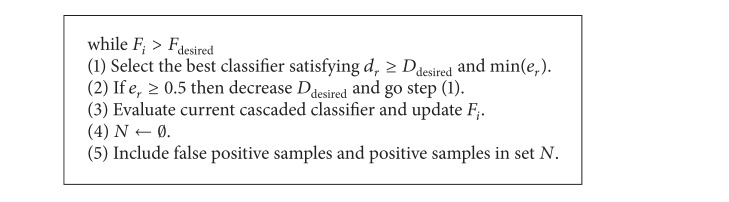
Classifier algorithm.

**Algorithm 3:**
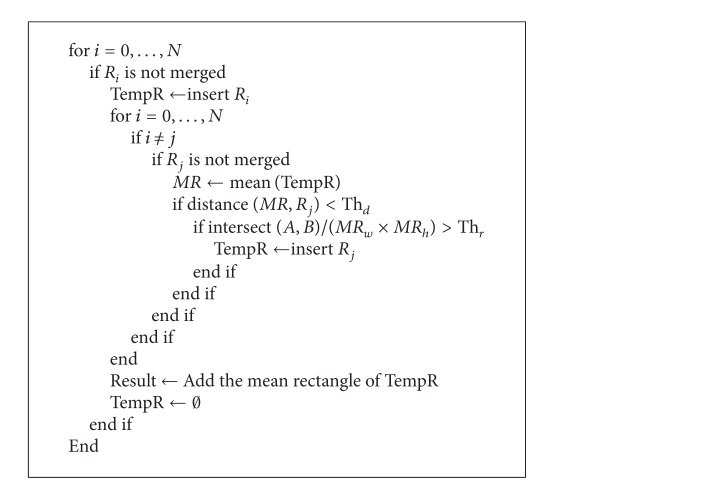
Merge algorithm.

**Algorithm 4:**
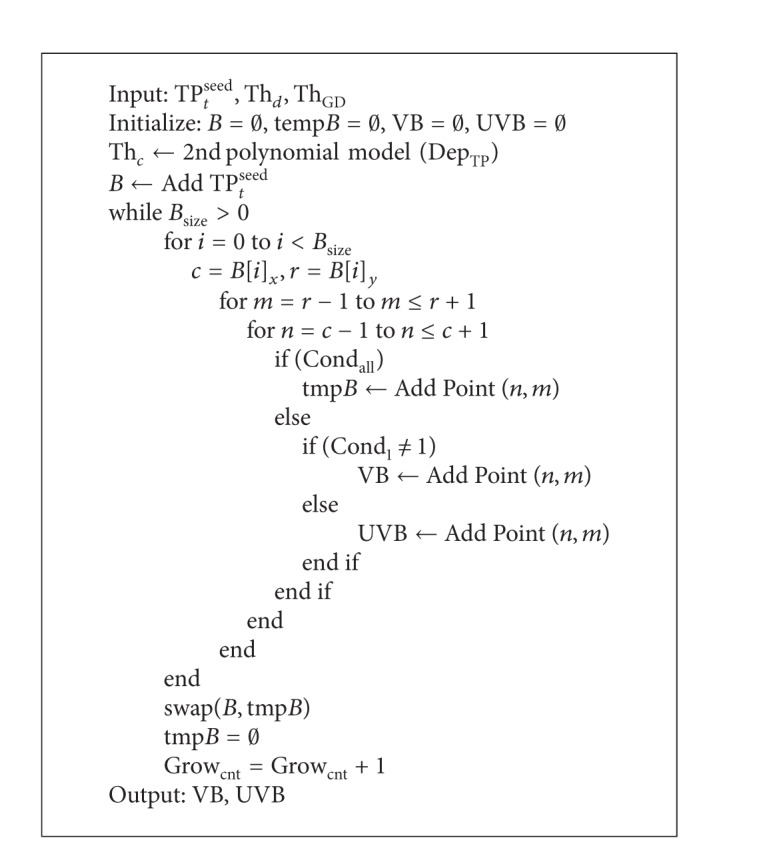
Algorithm for region growth.

**Table 1:** Comparison of detection rate and false positive rate of various AdaBoost algorithms.

	MFPR (maximum acceptable false positive rate)							
	0.7	0.6	0.5	0.4	0.3	0.2	0.1	0
Discrete AdaBoost								
DR	0.96	0.99	0.98	0.99	1	0.97	0.99	0.96
FDR	0.72	0.73	0.74	0.87	0.71	0.85	0.56	0.13
Computation (ms)	25.76	20.13	19.82	23.70	32.60	36.18	39.63	123.56
Real AdaBoost								
DR	0.98	0.99	0.97	0.96	**0.99**	0.99	1	0.94
FDR	0.47	0.39	0.27	0.45	**0.18**	0.26	0.20	0.01
Computation (ms)	15.63	15.77	15.42	16.85	**17.33**	21.36	24.18	82.83
Gentle AdaBoost								
DR	0.96	0.99	0.97	0.97	0.97	0.97	0.98	**0.98**
FDR	0.46	0.49	0.40	0.33	0.59	0.37	0.24	**0.01**
Computation (ms)	16.22	18.28	20.62	18.84	18.71	18.39	26.53	**86.15**
Proposed method								
DR	**0.97**							
FDR	**0**							
Computation (ms)	**4.19**							

**Table 2:** Average error of tracking with various distance conditions (UNIT: pixels).

	1 m	1.5 m	2 m
“a”	5.7883	4.2890	2.1928
“b”	4.3927	3.9835	2.3920
Spiral	4.6908	3.2395	2.9803

**Table 3:** Average error of tracking with various velocity conditions (UNIT: pixels).

	Slow	Normal	Fast
“a”	3.7559	4.1664	8.1443
“b”	2.3446	3.4423	3.3796

**Table 4:** Computing time (Unit: ms).

	Detection stage	Tracking stage
Computing time	5.85	25.96
